# Beyond the groin: a retroperitoneal liposarcoma with an unusual presentation: a case report

**DOI:** 10.1097/RC9.0000000000001209

**Published:** 2026-10-22

**Authors:** Waleed Ahmad, Afza Saleem, Asma Saleem, Mohammed Hammad Jaber Amin

**Affiliations:** a Department of General Surgery, Ghurki Trust Teaching Hospital, Lahore, Pakistan; b Department of General Surgery, CMH Lahore Medical and Dental College, Lahore, Pakistan; c Department of Medicine, Alzaiem Alazhari University, Khartoum, Sudan

**Keywords:** case report, inguinal hernia, retroperitoneal liposarcoma, soft tissue sarcoma, spermatic cord, surgical excision

## Abstract

**Background:**

Retroperitoneal liposarcoma is an uncommon malignancy that can present with aberrant manifestations and can sometimes resemble an inguinal hernia, creating diagnostic difficulties.

**Presentation of case:**

We describe the case of a 52-year-old man who presented with a long-standing right inguinal swelling that was initially thought to be an inguinal hernia. Computed tomography with contrast enhancement showed a sizable retroperitoneal mass that extended into the inguinal canal, which was suspicious for malignancy. An exploratory laparotomy revealed a retroperitoneal tumor that followed the inguinal canal. Complete surgical excision was performed. A diagnosis of well-differentiated liposarcoma was established by histopathological and immunohistochemical analyses. The patient was referred for adjuvant radiotherapy and is in clinically stable condition, with no recurrence on follow-up.

**Discussion:**

Retroperitoneal liposarcomas are often asymptomatic and may grow to a considerable size before diagnosis. They rarely invade the inguinal canal and resemble more common benign pathologies, including inguinal hernias, which may delay proper intervention.

**Conclusion:**

This case highlights the importance of considering retroperitoneal malignancy in patients with atypical or non-reducible inguinal masses. Early imaging, correct diagnosis, and proper oncologic surgical planning are crucial to maximizing patient outcomes.

## Introduction

Soft tissue sarcomas are uncommon malignancies, representing only about 1% of all adult malignancies, with 10%–15% developing in the retroperitoneum^[^1^]^. Liposarcomas constitute the leading tumors of the retroperitoneum, accounting for at least 15% of all retroperitoneal tumors and up to 50% of all soft tissue sarcomas^[^2^]^. Retroperitoneal liposarcomas typically manifest in patients within the fifth to seventh decades of life, with an equal gender distribution. These tumors are well known for their indolent growth and ability to attain a large size before clinical diagnosis, and they occur in approximately 10%–36% of all adult soft tissue sarcomas^[^3^]^.

HIGHLIGHTS

- Retroperitoneal liposarcoma can rarely mimic inguinal hernias.
- Imaging is a key factor in distinguishing between benign and malignant inguinal masses.
- Early suspicion in the preoperative phase deters surgical mismanagement.
- Total surgical resection is the mainstay of therapy.

It is critical to make a diagnostic distinction between a primary liposarcoma of the spermatic cord and a retroperitoneal liposarcoma extending into the inguinoscrotal region because the latter requires more extensive surgical planning to achieve oncologically sound resection margins^[^4^]^.

It is noteworthy that poor primary surgical treatment, that is, simple hernia repair as opposed to an oncologic resection, may cause a recurrence with a poor prognosis^[^4^]^.

We present a rare case of retroperitoneal liposarcoma that spread to the inguinal region and first presented as an inguinal hernia. This report emphasizes the importance of preoperatively establishing the retroperitoneal origin of a tumor using cross-sectional imaging to avoid accidental local repair of hernias, which may result in tumor violation, intra-operative capsular rupture, and poor oncological control. This case report is presented in line with the SCARE 2025 criteria^[^5^]^.

## Presentation of case

The patient is a 52-year-old married man with no other major medical or surgical history but with a 2-year history of steadily progressive swelling in the right inguinal region, with noted heaviness and occasional pain. He had no history of smoking or drinking. The patient had no known allergies, and his family history was unremarkable. There were no associated urinary or bowel symptoms. Importantly, the extreme chronicity (2 years), the lack of acute severe tenderness and overlying skin changes, the complete absence of bowel or urinary obstructive symptoms, and the clinical non-reducibility without evidence of strangulation were all important diagnostic warning signs that this was an atypical retroperitoneal process rather than an acute incarcerated inguinal hernia. Before presentation to our facility, he was evaluated at another hospital 1 week earlier, where an abdominal ultrasound examination indicated a suspicious intra-abdominal mass, and thus further investigation was pursued.

A large, well-circumscribed mass, measuring approximately 26 × 16 × 11 cm and predominantly fat-attenuating, was seen on computed tomography (CT), making the diagnosis of retroperitoneal liposarcoma very likely, with the mass extending directly through the internal inguinal ring into the inguinal canal. The mass had thin, irregular internal septations (<2 mm), did not contain solid, nodular, or calcific components, and showed minimal heterogeneous contrast enhancement. It had a distinct mass effect, displacing the cecum and ascending colon toward the anterior and medial parts of the abdomen, compressing the right ureter without grossly invading adjacent viscera, and causing peripheral displacement of the structures of the right spermatic cord.

Abdominal examination revealed a non-tender mass in the right hypochondrium and iliac fossa. Laboratory findings were normal except for mildly elevated gamma-glutamyl transferase (98 IU/L) and alanine transaminase (48 IU/L).

We acknowledge the diagnostic value of cross-sectional CT images in illustrating tumor continuity through the deep ring. Regrettably, owing to mandatory institutional data-purging protocols for historical electronic archives, original high-resolution DICOM files could not be retrieved for graphical publication. To compensate for this limitation, official radiology reports were verified and corroborated by operative findings and are described in detail below.

Since the history suggested a slowly increasing right inguinal swelling and imaging revealed a mass in the right iliac fossa, an irreducible inguinal hernia, a spermatic cord lipoma, or a liposarcoma based in the retroperitoneum were initially on the list of differential diagnoses. The atypical distribution pattern of contrast on contrast-enhanced CT gave rise to the suspicion that the process was malignant rather than benign, especially a liposarcoma rather than a lipomatous tumor.

High-resolution chest CT showed no signs of active disease or metastatic disease; only mild healed inflammatory changes were present. An exploratory laparotomy was planned for the patient. During surgery, a retroperitoneal mass measuring 26 × 16 × 11 cm, extending to the inferior extent of the inguinal canal, was seen (Fig. 1). Intraoperative handling was minimized to preserve tumor capsule integrity. The main retroperitoneal tumor mass and its inguinal extension were completely resected with negative macroscopic and microscopic margins (R0 clearance). To facilitate gross pathological evaluation and photography after excision, the specimen was sectioned *ex vivo*. At no point during the dissection was the tumor capsule violated. No adjacent organ resections were required. Complete macroscopic clearance was obtained, and the oncologic resection was later confirmed definitively by margin histopathology, which demonstrated microscopically clear margins.

**Figure 1.**
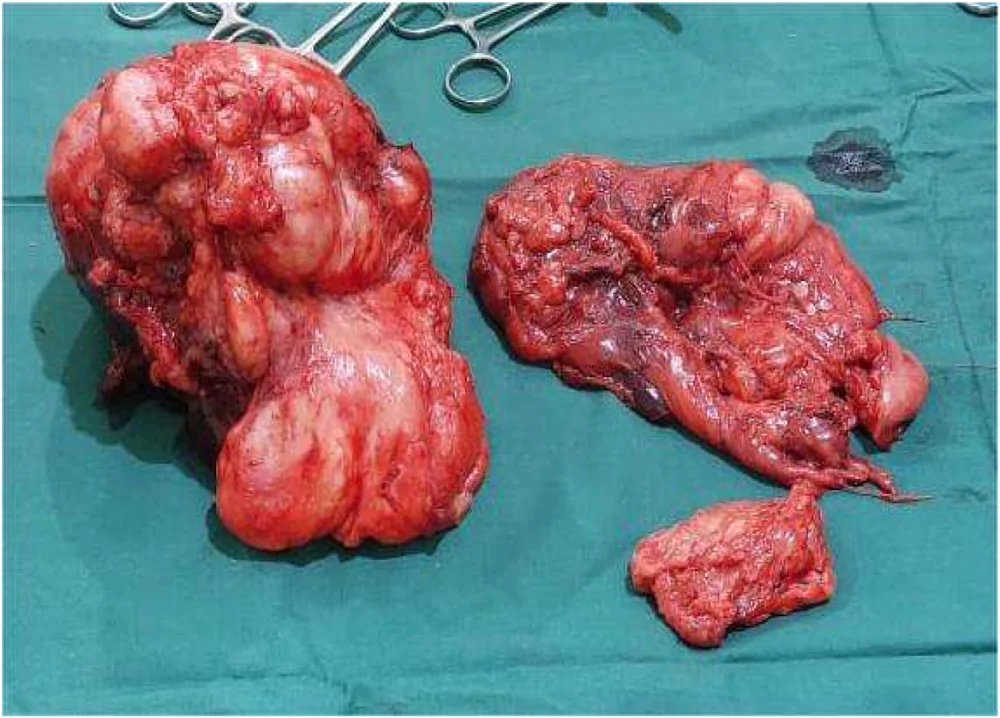
Gross macroscopic specimen of the excised retroperitoneal liposarcoma post-resection. The intact specimen achieved R0 clearance without intraoperative capsular violation and was sectioned *ex vivo* for gross anatomical display and histological processing.

Intraoperatively, the patient did not have a true concomitant indirect inguinal hernia sac containing bowel or omentum, but instead the large retroperitoneal mass acted as a pseudohernia sac, structurally dilating and extending through the deep inguinal ring into the canal. After the full delivery and mobilization of the inguinal component in one piece, the enlarged internal ring and canal floor were closed with interrupted polypropylene sutures (McVay technique) to prevent recurrence. No prosthetic mesh or material was used in accordance with oncological principles to avoid foreign-body contamination in a sarcoma bed.

Histopathology revealed a well-differentiated liposarcoma (WDLPS) composed of mature adipocytes with focal cytological atypia and occasional lipoblasts in fibrous septa, and focal myxoid stromal changes. There was no evidence of tumor necrosis or dedifferentiation. Mitotic activity was low (<1 mitosis per 10 HPF). Surgical margins were free of tumor. The diagnosis was confirmed by immunohistochemistry, which showed positivity for CD34 and negativity for SOX10, MUC4, CD117, and STAT6, in accordance with the WHO classification of WDLPS.

Although molecular techniques such as fluorescent *in situ* hybridization or nuclear immunohistochemistry for the detection of MDM2/CDK4 gene amplification are the best available diagnostic tools for distinguishing WDLPS from benign lipomas, the facilities for performing these tests were not available at our local treatment facility. Despite this, the classic morphological features (highly variable adipocyte size, clear nuclear atypia of stromal spindle-shaped cells in dense fibrous septa, and classic multi-vacuolated lipoblasts), together with the diffuse stromal CD34 positivity, confirmed the diagnosis.

The patient completed adjuvant radiotherapy without significant toxicity and remains under 6-month oncologic surveillance. At the 24-month follow-up, he remains clinically stable with no evidence of local or systemic recurrence.

## Discussion

Inguinal manifestations of retroperitoneal liposarcomas are rare (<6.6%), occurring when primary retroperitoneal tumors track down the deep inguinal ring into the canal ^[^6^]^. Per WHO criteria, liposarcomas are categorized into WDLPS, dedifferentiated liposarcomas (DDLPS), myxoid, pleomorphic, and myxoid pleomorphic subtypes^[^7^]^. WDLPS is locally invasive with low metastatic potential; however, our patient’s tumor exhibited classic low-grade WDLPS features.

When the condition occurs, the most frequent symptoms are non-specific abdominal pain, abdominal distention, or signs of nearby organ compression^[^8^]^. In our report, the patient had a chronic, painless right inguinal mass initially suspected to be an inguinal hernia. This finding is also reported in the literature, where most liposarcomas are initially misdiagnosed in the preoperative stage as benign inguinal hernias or lipomas^[^9^]^.

Radiological imaging is vital for diagnosis and staging. CT is the modality of choice to assess fat content, local invasion, and tumor size. CT findings in our case revealed a complex, fat-containing mass originating in the retroperitoneum that extended into the right iliac fossa and the inguinal canal. These types of presentations have been reported, with retroperitoneal disease extending to the pelvis and inguinal areas, permitting tumor spread along the testicular veins and into testicular structures within the spermatic cord^[^10^]^.

Our patient’s clinical course suggests a slow progression over 2 years, without any metastases at the time of diagnosis, which is typical of the WDLPS type. This is in contrast to DDLPS, which has been described as aggressive, with rapid growth, early local recurrence, and a high metastatic potential (often to the lungs). While WDLPS exhibits low mitotic activity (<1 mitosis per 10 HPF) and carries a favorable short-term prognosis regarding distant disease, its anatomical location within the retroperitoneum presents a high risk for persistent local recurrences (20%–90%) due to the challenges of achieving wide negative margins around vital retroperitoneal structures^[^11^]^. This underscores the immense diagnostic difficulty of slowly growing irreducible inguinal masses and the necessity of cross-sectional imaging prior to surgery to prevent inadvertent local inguinal hernia repair or incomplete removal ^[^5,10^]^.

Preintervention fine-needle aspiration biopsy or core-needle biopsy can sometimes be performed in the management of large retroperitoneal masses to confirm histological variants and to inform neoadjuvant planning. In presentations that mimic non-reducible or incarcerated hernias, however, primary biopsy is frequently avoided or contraindicated because of the need for immediate surgical correction to relieve mass-effect and the risk of needle-tract tumor seeding in the subcutaneous tissues of the groin.

The histological subtypes and surgical margins are significant prognostic factors^[^7^]^. The most important independent factor predictive of long-term outcome in retroperitoneal sarcomas is achievement of an R0 resection (microscopically negative margins). In our case, an en bloc tumor removal was achieved through exploratory laparotomy without evidence of intraoperative metastasis, which is the standard surgical approach to retroperitoneal liposarcoma and yields the best chance for long-term control of the tumor^[^7^]^. A multidisciplinary meeting was held to discuss the case further regarding postoperative management. Following multidisciplinary review, adjuvant external beam radiotherapy (EBRT; 50.4 Gy in 28 fractions) was delivered to the retroperitoneal and right inguinal fields. Although R0 clearance was obtained, tumors tracking through tight anatomical compartments carry a high risk of regional recurrence due to potential cell shedding during mobilization, justifying EBRT for local compartment control^[^12^]^.

## Conclusion

Retroperitoneal liposarcoma presenting as an inguinal hernia is a rare but critical differential diagnosis for atypical groin swellings. A high index of clinical suspicion and preoperative cross-sectional imaging can prevent inappropriate hernia repair. Complete en bloc surgical excision, combined with multidisciplinary planning and adjuvant therapy, optimizes long-term oncological outcomes.

## Data Availability

Within the article, the data used to support the findings of the present study are available. Any additional information is confidential and cannot be disclosed to the audience.
